# Participant Perspectives on a Community Health Worker Intervention to Reduce Infant Mortality: A Mixed Methods Assessment of the Bronx Healthy Start Partnership

**DOI:** 10.1007/s10995-024-04014-1

**Published:** 2024-11-22

**Authors:** Elisa M. Fisher, Alma Idehen, Luisa Cárdenas, David W. Lounsbury, Foram Jasani, Caryn R. R. Rodgers, Mayssa Gregoire, Rebecca Williams, Linda Weiss, A. Hal Strelnick

**Affiliations:** 1 Center for Evaluation and Applied Research, The New York Academy of Medicine, 1216 Fifth Ave, New York, NY 10029 USA; 2 Albert Einstein College of Medicine, 1300 Morris Park Avenue, Bronx, NY 10461 USA

**Keywords:** Community health workers, Home visiting, Pregnancy and childbirth, Social support, Program evaluation

## Abstract

**Introduction:**

Healthy Start is an initiative to reduce infant mortality and improve birth equity throughout the US, in large part by deploying community health workers (CHWs) to conduct home visits and provide educational and emotional support to new and expectant parents.

**Methods:**

A mixed-methods assessment of the Bronx Healthy Start Partnership (BxHSP) was conducted as part of a quality improvement initiative to understand client perspectives regarding the impact of BxHSP on short- and intermediate-term outcomes that affect long-term well-being. Phone interviews (*n* = 16) and online surveys (*n* = 62) were conducted in English and Spanish with BxHSP participants in 2020 and 2022. The interview sample was selected purposefully; interview participants were eligible if they gave birth prior to mid-March 2020 and had received at least one CHW home visit. All individuals with open BxHSP cases (*n* = 379) were invited to complete the survey.

**Results:**

Findings suggest that BxHSP CHWs can provide vital psychosocial, material, and educational resources that help engaged participants feel supported as new parents and develop knowledge and skills related to infant care. Results further suggest that these short-term outcomes contribute to lower stress, increased self-efficacy, and health-promoting infant care practices, enabling participants to feel more confident and capable as new parents.

**Discussion:**

Findings underscore how programs like BxHSP can help address gaps in resources and improve health and well-being for pregnant and postpartum participants. Limitations include possible selection, recall, and/or social desirability biases as response rates were low and data were self-reported and retrospective. Limitations were addressed in part through triangulation of qualitative and quantitative data.

## Significance

**What is already known?:**

Community health worker (CHW) programs for expectant and new parents have been found to improve birth outcomes, increase the likelihood of breastfeeding and following safe sleep practices, and improve utilization of pre- and postnatal care.

**What this study adds?:**

By providing participants with material and psychosocial support while fostering increased knowledge and skills related to childbirth and parenting, CHW programs can help to reduce stress, improve self-efficacy, and increase participation in health-promoting infant care practices among expectant and new parents. Findings provide important guidance that should inform program planning, improvement, and replication of CHW programs in the US.

## Introduction

The infant mortality rate in the United States (US) remains unacceptably high, in large part due to stark racial inequities in birth outcomes that persist across the country. As of 2018, the infant mortality rate (IMR) among infants born to Black mothers (10.8 deaths per 1000 live births) was more than twice the IMR of infants born to non-Hispanic white and Asian mothers (4.6 and 3.6 deaths per 1000 live births, respectively) (Jang & Lee, 2022). These disparities result from the chronic and toxic stress associated with experiences of racism and discrimination, as well as myriad structural inequities related to social and environmental drivers of health, such as healthcare quality, economic security, air quality, and more (Reno & Hyder, 2018).

Growing evidence suggests that community health worker (CHW) and home visiting programs may improve birth outcomes, particularly in marginalized communities that experience structural barriers to good health. Studies have linked participation in CHW programs during and after pregnancy to improved birth outcomes, including healthier birth weight and fewer and shorter stays in neonatal intensive care units, as well as increased knowledge, greater likelihood of breastfeeding and following safe sleep practices, and improved utilization of pre- and postnatal care (Cunningham et al., 2020; Gilmore & McAuliffe, 2013; Hitchcock et al., 2020; Pan et al., 2020; Scharff et al., 2022; Straughen et al., 2023).

In 1991, the Human Resources Services Administration (HRSA) established Healthy Start, a national initiative to reduce infant mortality in the US. A core feature of the initiative is deployment of CHWs who conduct home visits, provide education, and connect expectant and new parents to resources. In 2014, the Healthy Start program began placing a greater emphasis on the life-course model, which added a focus on intermediate health outcomes, recognizing their long-term impact on infant mortality rates (Lu & Johnson, 2014; Thomas et al., 2015).

In 2023, there were 101 Healthy Start programs across 37 states and the District of Columbia. In 2020, a national evaluation of Healthy Start programs found that participants had earlier initiation of and greater frequency of prenatal care, greater likelihood of following safe sleep practices, and more favorable birth weights (Hitchcock et al., 2020). However, this evaluation did not examine how program participation led to these outcomes.

The aim of the present study was to use mixed methods to examine client perspectives on how a Healthy Start program in the Bronx, New York, supports parent and child wellbeing during and after pregnancy. Understanding how and why CHW programs work will provide valuable insights related to program planning, improvement, and replication.

## Methods

An independent evaluator, used an exploratory sequential design (Fetters et al., 2013) to conduct a mixed methods evaluation to understand client perspectives on the Bronx Healthy Start Partnership (BxHSP) and its impact on short- and intermediate-term outcomes, which are expected to lead to longer-term well-being. Interviews were initially used to explore participant perspectives on program activities and outcomes. Findings from interviews were used to develop a survey assessing the extent to which a broader sample of participants agreed or disagreed with key interview themes. All activities were conducted in accordance with prevailing ethical principles and protocols were approved by NYAM’s Institutional Review Board. All participants consented to participation and received a gift card for participation.

### Program Description

Since 2014, BxHSP has been implementing Healthy Start to improve birth outcomes in the Bronx. With funding from HRSA, BxHSP employs CHWs who conduct outreach, home visits, and educational workshops; provide connections to community-based social and medical services; and offer one-on-one support to new and expectant parents.

**Fig. 1:**
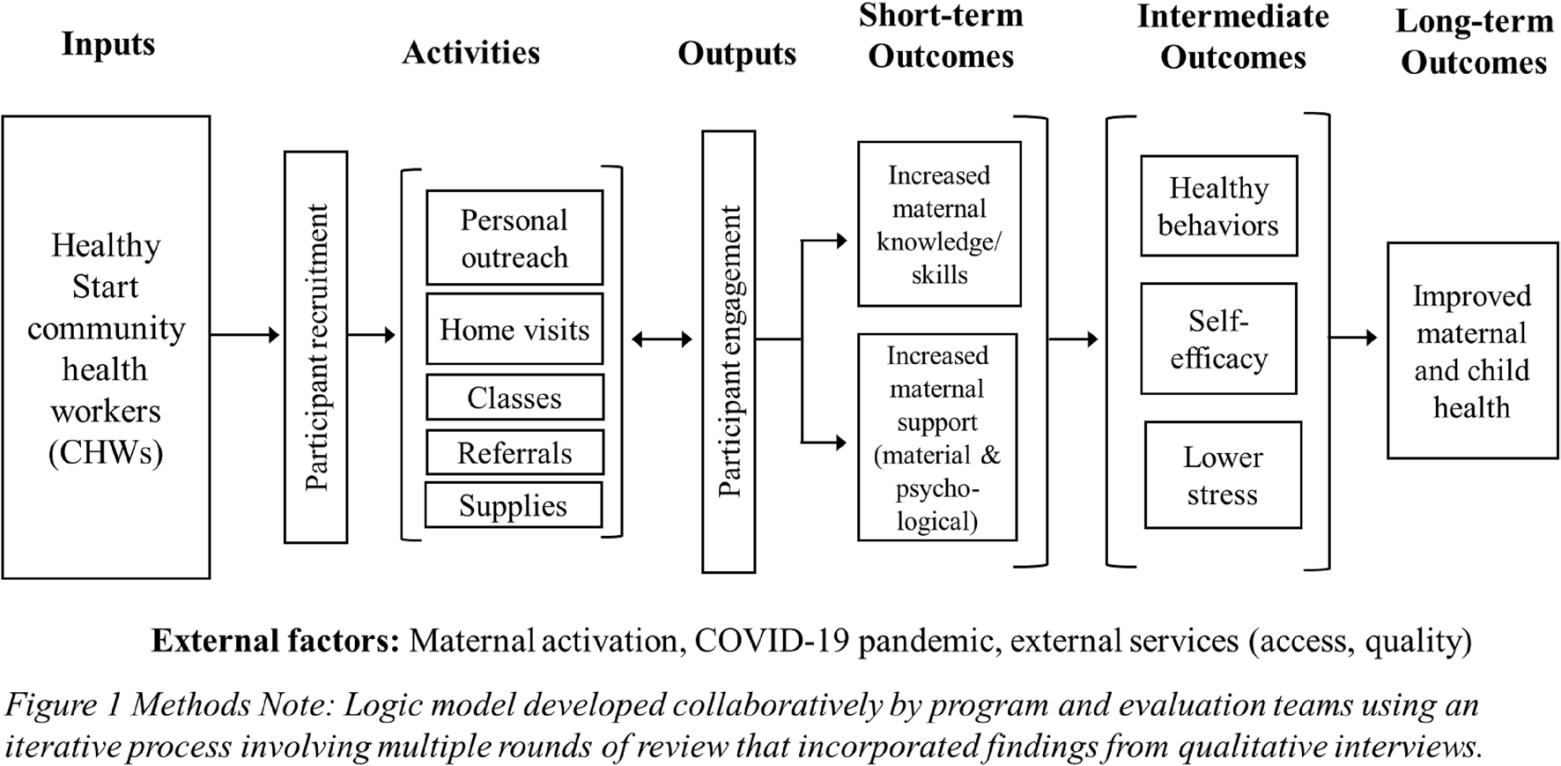
Bronx Healthy Start Partnership empirical logic model illustrates relationships between program inputs and activities, short- and intermediate-term outcomes (as described by study participants) and desired long-term outcomes

Figure 1 provides a visual representation of the program’s logic model, illustrating relationships between program inputs and activities, short- and intermediate-term outcomes—as described by study participants—and desired long-term outcomes.

### Qualitative Data Collection and Analysis

External evaluators conducted 16 semi-structured phone interviews with BxHSP participants between June and October 2020 in English and Spanish. The qualitative research team consisted of two evaluators trained in qualitative research who had no prior relationship to participants.

The interview sample was selected purposefully: BxHSP participants were eligible if they gave birth prior to March 12, 2020 and had completed at least one home visit with a CHW. Home visits, one of many ways that participants engaged with CHWs, were identified as a proxy for level of engagement, and engaging in at least one suggested that participants had sufficient experience to speak to program quality and impact.

Using de-identified participant data, evaluators selected a sample (*n* = 62) that was racially, ethnically, and linguistically similar to the overall BxHSP participant population. CHWs then contacted selected participants to provide information on interviews and request permission to share contact information with evaluators. Evaluators then reached out to interested participants (*n* = 31) by phone to share additional details and schedule the interview. A total of 16 interviews were completed; the remaining 15 individuals were either unreachable by phone or did not join their scheduled interview.

Interviewees were informed that interviewers worked at NYAM  and were partnering with BxHSP on an evaluation exploring clients’ perceptions of BxHSP services, BxHSP impact, and recommendations for improvement. Interviews were audio-recorded and professionally translated (if conducted in Spanish) and transcribed. They ranged in length from 36 to 68 min (*mean* = *50.25*).

Transcriptions were maintained, coded by the qualitative research team, and analyzed using content analysis in NVivo 12. The codebook was developed, refined, and applied using an iterative process, and included both pre-identified topics and those that emerged from the data. Findings were presented to the BxHSP Community Action Network, consisting of BxHSP participants, CHWs and community leaders, who were invited to provide input on findings, which were then used to develop a data-driven logic model.

### Quantitative Data Collection and Analysis

After interview analyses were complete, a participant survey was conducted to examine alignment of program perceptions across a broader sample of BxHSP participants. Survey questions were informed by interview findings.

All individuals with open cases in BxHSP’s data system (*n* = 379) as of October 31, 2022 were invited to complete the confidential survey in English or Spanish. Surveys were self-administered online via Qualtrics, a web-based survey platform. Between November 29 and December 20, 2022, four outreach emails were sent to eligible participants by the evaluators. Outreach emails provided information on the survey, including its purpose, the fact that it was confidential, and details on incentives. CHWs also reached out to clients one to three times via phone or text message to encourage participation. Participants were informed that the survey was voluntary and confidential and that CHWs would not know who completed the survey.

Sixty-two BxHSP participants completed the survey, none of whom had completed an interview. Survey data were downloaded and transferred to STATA SE (version 15; College Station, TX) for cleaning, management, and analysis. Descriptive statistics were generated for all variables.

### Integration of Qualitative and Quantitative Data

Evaluation team members compared findings from the survey to key themes from interviews to assess similarities and differences in participants’ perspectives on the quality of experience and outcomes of participating in BxHSP.

## Results

### Participant Characteristics

Table 1 displays demographic and background information for interview and survey participants. Forty-four percent of interviewees and 38% of survey respondents identified as Black or African American; 38% of interviewees and 35% of survey respondents identified as Hispanic or Latinx/e. One quarter (25%) of those interviewed and nearly one-fifth (19%) of those surveyed responded in Spanish.

**Table 1:** Interview and survey participant demographic information

Characteristics		Interview participants		Survey participants	
		*n*	(%)	*n*	(%)
Age					
	18–24	4	(25)	8	(13)
	25–34	4	(25)	36	(58)
	35–44	6	(38)	18	(29)
	Missing	2	(13)	0	(0)
Gender					
	Woman	16	(100)	62	(100)
Race/ethnicity					
	Black or African American	7	(44)	22	(35)
	Hispanic or Latinx/e	6	(38)	40	(65)
	Multiracial or Multiethnic^a^	2	(13)	-	-
	Native Hawaiian or Pacific Islander	1	(6)	1	(2)
	Indigenous American or Alaska Native	1	(6)	1	(2)
	White	1	(6)	5	(8)
	Asian or Asian American	0	(0)	4	(6)
	Missing	3	(19)	0	(0)
Language^b^					
	English	12	(75)	50	(81)
	Spanish	4	(25)	12	(19)

* n* = 16 for interview participants and n = 62 for survey participants

^a^Demographic information for interview participants relied on program records, which used mutually exclusive racial categories and a separate question on ethnicity (Hispanic/Non-Hispanic); interviewees who identified with more than one racial or ethnic group selected "multiracial/multiethnic." Surveys included a demographic questionnaire that allowed participants to select multiple racial/ethnic identities that were not mutually exclusive; multiracial/multiethnic was not a survey response option

^b^Refers to the language in which the interview or survey was completed

### Short-Term Program Outcomes

Interviewees valued their relationships with CHWs and the consistent psychosocial support provided. They explained that CHWs communicated with them regularly via phone, text message, and home visits; encouraged them to attend educational workshops; and offered service referrals, parenting education, and support. Interviewees emphasized the importance of having a person available to listen, validate their concerns, and problem solve, particularly in the early stages of parenting.

[The CHW explained] “I may not be in the home with you 24/7, but I'm always reachable. You can always come to the office. I can always make a home visit. I can attend to you when you have an emergency as your social worker.” And that is, you know, amazing in itself. (English-speaking participant)

[My CHW] was wonderful and of great help, because they give you a lot of information, they help you a lot, they listen to you. That’s what’s most important when you’re a single mother who has just given birth, you need someone to listen to you a lot. (Spanish-speaking participant)

Interviewees also reported that CHWs helped them access resources by connecting them to external community-based services, including nutrition assistance programs, mental health counseling, doulas, and health insurance. CHWs also provided participants with free infant care supplies (e.g., diapers, formula, cribs).

I told [the CHW] that I needed health insurance. She did give me the information to call so I could register, or the link. Then, I needed to ask her about the health insurance again, because, I think, they denied me the first time… she said, “You need to call them back and tell them there’s been a change in your salary,” so I did. Then I got it fixed and they mailed me my health insurance card for the baby and me. (English-speaking participant)

Survey results corroborate interview findings: respondents agreed that CHWs made them feel supported (89%); treated them with respect (89%); were easy to reach (87%); and communicated effectively (85%). Survey respondents also noted that participation in BxHSP helped them access social or community services (79%) (Table 2). Fifty percent of survey respondents reported that the program helped them get supplies, such as diapers and cribs (data not shown).

**Table 2:** Self-reported short-term program outcomes for participants in BxHSP

Short-term outcomes	n*	(%)
Participation in BxHSP…		
…Made me feel supported as a parent	54	(87)
…Provided me with valuable parenting skills	50	(81)
…Helped me access social or community services	49	(79)
…Taught me to put the baby to sleep safely	46	(74)
Staff in BxHSP…		
…Treated me with respect	55	(89)
…Made me feel supported	55	(89)
…Were easy to reach	54	(87)
…Communicated with me effectively	53	(85)
…Taught me important lessons	49	(79)

*Note*. Data source: Participant survey (*n* = 62). Items assessed on a 4-point agree-disagree Likert scale; no items had more than one missing response

*Includes those who somewhat or strongly agree

Interviewees consistently attributed improved knowledge and skills around pregnancy and newborn care to program participation. They described valuable lessons related to breastfeeding, safe infant sleep practices, child nutrition, and contraception.

[BxHSP] also explained about SIDS in the class, and they gave different information about that, explaining what it is and what the causes are—not really the causes, because it happens sometimes for no reason, but what you can do to prevent it. (English-speaking participant)

Most survey respondents agreed that participation in BxHSP provided them with valuable parenting skills (81%) and that CHWs taught them important lessons (79%) (Table 2).

### Intermediate Program Outcomes

Interview participants attributed changes in infant care practices to their participation in BxHSP.  They altered infants’ sleep environments based on lessons learned through the program and linked their motivation and ability to breastfeed to workshops and tailored lactation support from CHWs. Nearly three quarters (71%) of survey respondents agreed that BxHSP improved their ability to breastfeed (Table 3).

I wasn't specifically planning to breastfeed… and it was thanks to those [BxHSP] classes, which were the only ones I took. And honestly, the information was so good, so clear, so precise, so motivating that honestly, I have been breastfeeding exclusively now for 16 months. (Spanish-speaking participant)

**Table 3:** Self-reported intermediate program outcomes for participants in BxHSP

Intermediate outcomes		n*	(%)
Participation in BxHSP…			
	…Helped me feel more prepared for childbirth	47	(76)
	…Increased my self-confidence as a parent	46	(74)
	…Helped me feel less stressed	46	(74)
	…Improved my ability to breastfeed	44	(71)
Because of my participation in BxHSP…			
	…I am more comfortable standing up for my family with healthcare providers	47	(76)
	…My children are healthier	45	(73)
…	…I am healthier	43	(69)
	…My mental health has improved	41	(66)

*Note.* Data source: Participant survey (*n* = 62). Items assessed on a 4-point agree-disagree Likert scale; no more than one missing response for any given item

*Includes those who somewhat or strongly agree

According to interviewees, greater access to psychosocial and material supports, and increased knowledge and skills resulting from program participation, enabled them to feel more confident and capable as new parents.

Now that I know different [breastfeeding] positions and... what a correct latch is and what is not, then I feel like I’m better…I feel, like, more comfortable with what I’m doing. Like I feel like I’m doing it correctly. (English-speaking participant)

Interviewees also reported that the program equipped them with the information and confidence to effectively advocate for themselves and their children within healthcare settings, especially around topics related to childbirth and child development.

[My CHW] explained to us when you first receive the baby, or you go to the hospital, you have a right as a patient and as the mother to say, “I don’t want this…You shouldn’t stay quiet, because you know how your body is experiencing the process, and everything you’re going through… Now I say, should I have another opportunity, I will speak up. (English-speaking participant)

Approximately three quarters of survey respondents agreed that participation in the program increased their self-confidence as a parent, helped them feel more prepared for childbirth, and helped them feel more comfortable standing up for themselves or their child(ren) with healthcare providers (74, 76, and 76%, respectively).

Both survey respondents and interviewees reported that BxHSP contributed to lower levels of stress. Interviewees described the connection between greater access to resources, including social support from CHWs, and reductions in stress associated with self-doubt, social isolation, and limited access to financial resources.

Sitting with a [BxHSP CHW] was like sitting with a friend and having a conversation and forgetting a little bit about the problems, the stress of the house, the stress of the children... If today I am strong and have been able to keep going, it is very much because of this program. (Spanish-speaking participant)

I overthink things too much, and it's like “how am I going to do this, how am I going to do that?” So, [my CHW] was the person of resources and saying “Okay, well, we can help you with diapers. We can help you find clothes. We can help you get a crib, if that's what you're stressing about.” And it was able to kind of clear my head a little bit more. (English-speaking participant)

Survey respondents agreed that program participation helped them feel less stressed (74%) and led to improved overall health (69%), mental health (66%), and child health (73%).

## Discussion

Findings from this evaluation suggest that BxHSP CHWs provide psychosocial, material, and educational resources that can help engaged participants feel supported as new parents and develop knowledge and skills related to infant care and parenting. Results further suggest that these short-term outcomes can contribute to intermediate outcomes, including: (1) lower stress, (2) greater levels of self-efficacy, including comfort advocating for their families in healthcare settings, and (3) health-promoting infant care practices. A strong body of literature provides evidence that these intermediate outcomes impact long-term health (Hobel et al., 2008; Moon et al., 2022; Tilden et al., 2016).

Relief from stress was consistently highlighted as a key mechanism through which BxHSP impacts well-being. Though multiple program components contributed to lower stress levels, participants most frequently emphasized the importance of social support from CHWs. This finding is consistent with evidence that access to social support during pregnancy and the postpartum period is protective for mental health and promotes positive birth outcomes (Bedaso et al., 2021; Leahy-Warren et al., 2012; Mundorf et al., 2018; Pao et al., 2019; Stuart-Parrigon & Stuart, 2014).

Study participants also reported that participation in BxHSP contributed to greater self-efficacy related to parenting. Parenting self-efficacy is associated with improved perinatal outcomes, parent–child relationships, parental mental health, and child development (Albanese et al., 2019; Tilden et al., 2016).

Participants reported more comfort advocating for themselves and their children in healthcare settings, which may increase willingness to actively engage in care (Hibbard et al., 2005). Active patient engagement is recognized as an important strategy for improving care quality and reducing medical errors (Agency for Healthcare Research & Quality, 2022; World Health Organization, 2016). Increasing confidence and comfort with self-advocacy during and after pregnancy may be particularly valuable for Black women, who too often experience discrimination and mistreatment in healthcare settings (Vedam et al., 2019). While the onus to change is on the health systems and providers who fail to provide Black patients with appropriate care, Black mothers interviewed by Smith et al. suggested that the ability to self-advocate can be an important coping mechanism for enduring and addressing mistreatment and medical racism (Smith et al., 2022).

This evaluation had several limitations. Originally developed for quality improvement, data were self-reported and collected retrospectively. Response rates were low: approximately 25% for the interviews and 16% for the surveys. Thus, findings cannot be generalized to the entire population of BxHSP participants; instead, they are applicable to the participants who chose to complete an interview or survey, who may be more engaged or have had more favorable program experiences.

It is worth noting that engagement in Healthy Start may be motivated by a need for material support (e.g., diapers, formula) among new parents raising young children on low incomes, and that individuals, particularly those of color, may be hesitant to use the range of services offered due to a distrust of public health programs that have historically marginalized and criminalized their communities. As a result of this and myriad other stressors faced by new parents, there may be a constituency of participants who are minimally engaged with the program whose views are not represented in our findings.

BxHSP makes concerted efforts to build trust within the community and provide culturally appropriate, client-centered services by (1) engaging their Community Action Network, made up of at least 25% current or former participants, and (2) employing CHWs who are members of the communities being served. Still, program and study conditions likely introduced selection, recall, and/or social desirability biases. Limitations were, in part, addressed through engagement with researchers external to the program and use of mixed methods, allowing for triangulation of qualitative and quantitative findings.

The arrival of the COVID-19 pandemic was also a limitation. Beginning in March 2020, New York City enforced strict social distancing measures to control the spread of the virus, and BxHSP significantly altered its services, shifting home visits and educational workshops online. We were unable to account for variation in participants’ experiences with the program and its services that resulted from the emergency shutdown. While this did not affect data collection, it may have affected decisions around participation in evaluation activities.

## Conclusions

Evaluation participants confirm that engagement in BxHSP improves health and well-being during and after pregnancy. Sustained racial disparities in birth outcomes call for initiatives such as BxHSP that address inequitable conditions both within and outside of the healthcare system. Engaging pregnant and new parents in Healthy Start and similar CHW programs can help address gaps in resources and improve long-term outcomes by providing participants with the educational, material, and psychosocial supports they need to engage in recommended health behaviors, increase parental self-efficacy, and moderate the impact of common stressors during pregnancy and postpartum.

## Data Availability

Available upon request.

## Code Availability

Available upon request (Stata SE).
